# Persistent Anemia in an HIV Patient: The Role of Parvovirus B19

**DOI:** 10.7759/cureus.103168

**Published:** 2026-02-07

**Authors:** Priscila M Fiallo, Larry M Bush, Maria T Vazquez-Pertejo

**Affiliations:** 1 Department of Internal Medicine, Wellington Regional Medical Center, Wellington, USA; 2 Department of Medicine, Charles E. Schmidt College of Medicine, Florida Atlantic University, Boca Raton, USA; 3 Department of Pathology and Laboratory Medicine, Wellington Regional Medical Center, Wellington, USA

**Keywords:** acquired aplastic anemia, hiv-positive, parvovirus b-19, pure red cell aplasia (prca), viral infection-associated aplastic anemia

## Abstract

Parvovirus B19 (B19V) is a common single-stranded DNA virus that infects humans and is its only host. Although infection is often asymptomatic, B19V can cause significant hematologic disease due to its tropism for erythroid progenitor cells. Immunocompromised individuals, including those with human immunodeficiency virus (HIV), are susceptible to persistent infection and may develop pure red cell aplasia (PRCA) and chronic anemia. Since B19V infection is not routinely monitored in the United States, and its symptoms can overlap with other causes of anemia in HIV, diagnosis may be overlooked. Early identification is critical, as intravenous immunoglobulin (IVIG) can rapidly restore erythropoiesis and reverse transfusion dependence.

## Introduction

Parvovirus B19 is a single-stranded DNA virus member of the Parvoviridae family, having humans as the only known host [1]. Transmission of infection is primarily via the respiratory route, but may also occur through blood or blood products, as well as through organ transplantation. While approximately 50% of children have serologic evidence of infection, this figure reaches 80% in the elderly population, as the acquisition of infection continues throughout adolescence into adulthood [2]. Infection with B19V is not a notifiable disease in the United States and is not routinely surveilled; however, a rise in reported cases has been observed in 2024. A large portion of those infected remain asymptomatic or subclinical. Nevertheless, dependent on the immunologic and hematologic status of the host, infected individuals may present with a wide variety of disease manifestations [3].

Erythema infectiosum, more commonly referred to as the fifth disease or “slapped cheek” disease, is the most common childhood presentation. Infection during pregnancy may lead to fetal death in utero, hydrops fetalis, or, rarely, the development of congenital anemia. In the immunocompromised host, B19V can cause transient aplastic anemia. Persistent viremia can result in pure red cell aplasia (PRCA) and chronic anemia [3,4]. The prevalence of B19V-induced anemia in HIV-seropositive patients is probably higher than that generally recognized [5]. We report a case of severe, transfusion-dependent anemia in an HIV-infected adult ultimately diagnosed with B19V-associated PRCA, highlighting the importance of maintaining clinical suspicion for this treatable condition.

## Case presentation

**Figure 1:**
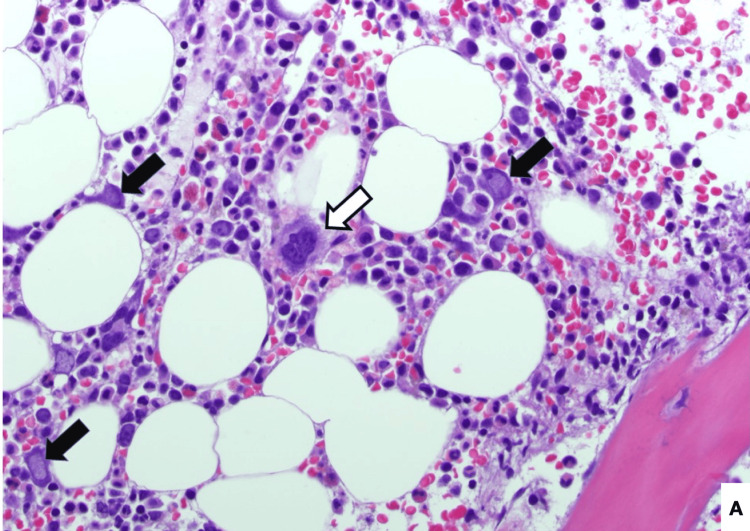
Bone marrow bone core biopsy, 200x, Hematoxylin and eosin (H&E) stain. B19V-infected erythroid precursor cells (black arrows indicate intranuclear viral inclusions; white arrows indicate megakariocytes for comparison).

**Figure 2:**
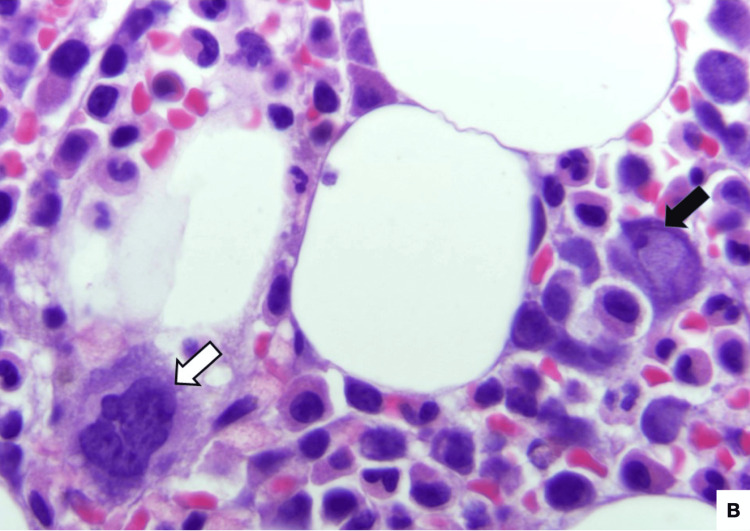
Bone marrow aspirate, 400x, Hematoxylin and eosin (H&E) stain. B19V-infected erythroid precursor cell (black arrow points to an intranuclear viral inclusion; white arrow indicates a megakaryocyte for comparison).

**Figure 3:**
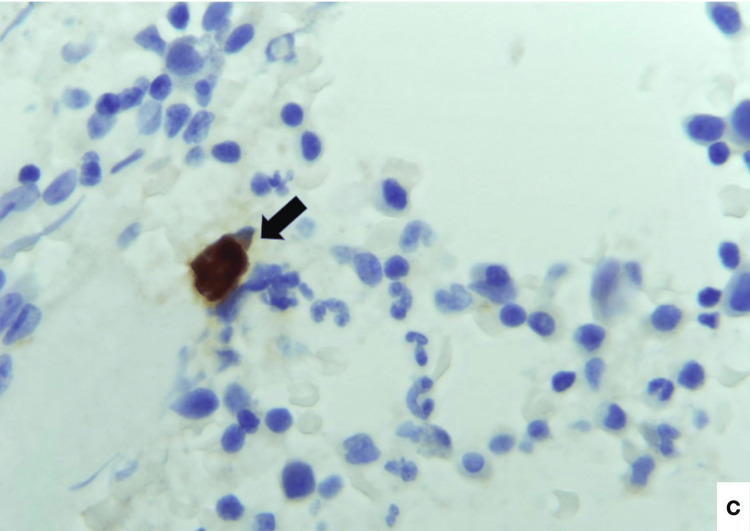
Bone marrow aspirate biopsy, 400x, B19V immunohistochemical stain - positive stain in an infected erythroid precursor cell (black arrow).

An asymptomatic 37-year-old HIV-infected man was referred to the hospital due to severe anemia. Physical examination was unremarkable except for pale conjunctivae. On admission, the hematogram revealed hemoglobin (Hgb) of 4.7 g/dl, hematocrit (HCT) of 13%, mean corpuscular volume (MCV) of 80 fl, white blood cell count (WBC) of 4.01x10³/μl, and platelets (PLTS) of 312x10³/μl (Table 1). He had a history of chronic anemia requiring transfusions, the etiology of which had not been previously determined. His CD4 T-cell lymphocyte count was 232 cells/ml (14.5%) with an HIV RNA load of 13,500 copies while being treated with bictegravir/emtricitabine/tenofovir-alfenamide. He tested positive for immunoglobulin M (IgM) but negative for IgG anti-B19V antibodies (Table 1). Quantitative, real-time polymerase chain reaction (PCR) testing for parvovirus B19 DNA was not performed due to a lack of availability. A bone marrow biopsy revealed marked erythroid hypoplasia with scattered large, atypical cells displaying abnormal erythroblast morphology (Figures 1, 2). These cells contained prominent ground-glass intranuclear viral inclusions, which stained positive for B19V by immunohistochemistry (Figure 3), supporting the diagnosis of B19V-associated PRCA.

**Table 1:** Laboratory tests during hospital admission. RBC: Red blood cells; WBC: white blood cells: Hgb: hemoglobin; HCT: hematocrit; PLT: platelets; MCV: mean corpuscular volume; MCH: mean corpuscular hemoglobin; MCHC: mean corpuscular hemoglobin concentration; RDW-SD: red blood cell - standard deviation; RDW-CV: red blood cell - coefficient of variation; MPV: mean platelet volume; TIBC: total iron binding capacity; CO_2_: carbon dioxide; AGAP: anion gap; BUN: blood urea nitrogen; eGFR: estimated glomerular filtration rate; PCR: polymerase chain reaction; Ig; immunoglobulin.

Laboratory (units)	Normal Ranges	Day 1 of admission	Day 2 of admission	Day 3 of admission	Day 4 of admission	Day of discharge
Hematology						
WBC (x10^3^/µL)	4.5-10.5	4.01	4.98	5.19	6.21	5.28
RBC (x10^6^/µL)	4.4-6.15	1.72	2.11	2.73	2.44	2.73
Hgb (g/dL)	14-18	4.7	6.1	7.6	6.9	7.9
Hct (%)	40-54	13.8	16.6	21.6	19.6	21.9
MCV (fL)	81-96	80.2	78.7	79.1	80.3	80.2
MCH (pg)	27-34	27.3	28.9	27.8	28.3	28.9
MCHC (g/dL)	32-36	34.1	36.7	35.2	35.2	36.1
RDW-SD (fL)	36-50	50.5	47.5	48.8	50.3	49
RDW-CV (%)	11-14.5	17.5	16.8	16.8	17.2	16.6
Platelets (x10^3^/µL)	150-450	312	283	316	325	282
MPV (fL)	6.9-10.5	10.3	10.3	9.9	10.8	9.9
		Received transfusion	Received transfusion		Received transfusion	
Chemistry						
Iron (µg/dL)	65-175	197				
TIBC calculated (µg/dL)	250-450	191				
Transferrin (mg/dL)	202-364	153				
Ferritin (mg/dL)	8-388	925				
Glucose (mg/dL)	74-106	96	94	98	88	108
Sodium (mmol/L)	134-148	141	140	141	141	139
Potassium (mmol/L)	3.6-5.2	3.3	3.8	3.8	3.6	3.7
Chloride (mmol/L)	95-110	110	113	11	112	109
CO_2_ (mEq/L)	21-32	28	25	25	23	28
AGAP (mmol/L)	5-15	6.3	6.8	8.8	9.6	5.7
BUN (mg/dL)	7-18	11	13	10	10	8
Creatinine (mg/dL)	0.7-1.3	1.07	0.86	0.95	0.81	0.9
BUN/Creat Ratio	N/A	10	15	11	12	9
Calcium (mg/dL)	8.5-10.1	8.7	8.6	8.4	8.1	8.8
eGFR Cr (mL/min/1.732)	N/A	92		106	116	112
Others						
HIV-1 RNA by PCR (copy/mL)	N/A	13500				
log10 HIV-1 RNA (copy/mL)	N/A	4.13				
% CD-4 (%)	30.8-58.5	14.5				
Absolute CD-4 (cells/µL)	359-1519	232				
Parvovirus IgM Index	0.0-0.8	2.1				
Parvovirus IgG Index	0.0-0.8	0.5				

## Discussion

Although rare in HIV-infected patients, B19V-related anemia is likely underdiagnosed [5]. Persistent parvovirus infection in such individuals is due to inadequate neutralizing antibody production secondary to a poorly functioning adaptive immune response. Adequate IgM and IgG antibody production is required for the elimination of B19V. The role of the cellular immune response in limiting B19V infection has been studied less intensively [6,7]. Despite antiretroviral therapy, our patient’s elevated HIV viral load and reduced CD4 T-cell percentage suggest non-compliance with his regimen [5]. Diagnosis requires clinical suspicion and is supported by the detection of anti-B19V antibodies along with B19V DNA PCR. Since viral isolation is not reliable, confirmation relies on the use of specific immunohistochemical stains to validate the presence of B19V in erythroid precursor cells. IVIG is the treatment of choice in severe cases. If relapse occurs less than six months after the initial treatment, an empirical maintenance treatment with a single-day infusion of 0.4 g/kg IVIG every four weeks may control the B19V viremia [8,9].

## Conclusions

Parvovirus B19 is an underdiagnosed yet treatable cause of severe anemia in immunocompromised patients, including those with HIV. To diagnose this condition, clinical suspicion should be supported by serological testing, and, if available, PCR testing and immunohistochemical staining for confirmation. Prompt recognition and management are key to preventing ongoing transfusion dependence and optimizing patient care. Therefore, increased awareness of Parvovirus B19 in immunocompromised populations is essential for healthcare professionals.
